# Case Report: Left ventricular hemorrhage associated with early neonatal mortality in multi-gene engineered cloned pigs

**DOI:** 10.3389/fvets.2026.1833278

**Published:** 2026-06-10

**Authors:** Dong-Hwan Kim, Dong-Eon Kim, Bumseok Kim, In-Sul Hwang, Geumyoung Sok, Seongsoo Hwang, Jeongwoong Lee

**Affiliations:** 1 Department of Animal Science, College of Natural Resources and Life Science, Pusan National University, Miryang, Republic of Korea; 2 Green Bio Science Major, School of Applied Life and Convergence Science, Pusan National University, Yangsan, Republic of Korea; 3 Biotherapeutics Translational Research Center, Korea Research Institute of Bioscience and Biotechnology, Daejeon, Republic of Korea; 4 Biosafety Research Institute and College of Veterinary Medicine, Jeonbuk National University, Iksan, Republic of Korea; 5 Columbia Center for Translational Immunology, Columbia University Irving Medical Center, Columbia University, New York, NY, United States; 6 National Institute of Animal Science, Rural Development Administration, Wanju, Jeollabuk-do, Republic of Korea

**Keywords:** cardiac hemorrhage, genetically modified pig, neonatal death, somatic cell nuclear transfer, xenotransplantation

## Abstract

Advances in genetic engineering have enabled the development of highly modified porcine models for xenotransplantation. Among available technologies, somatic cell nuclear transfer (SCNT) remains the most reliable method for generating multi-genic pigs carrying complex genetic modifications, including the knockout of porcine xenoantigens and the insertion of human immune regulatory genes. Despite these advances, the physiological viability of highly engineered cloned animals remains a major challenge. In our previous study, we generated cloned pigs carrying a knockout of α-1,3-galactosyltransferase (α-gal) and knock-in of five human immune-related genes: human decay-accelerating factor (hDAF), CD39 (hCD39), tissue factor pathway inhibitor (hTFPI), C1 inhibitor (hC1INH), and TNF-α-induced protein 3 (hTNFAIP3). Although genomic integration and cellular expression of these transgenes were confirmed, all piglets in this cohort died within 48 h after birth. Necropsy revealed no evidence of external trauma or systemic abnormalities in major organs. However, hemorrhagic lesions were observed in the left ventricular myocardium of all necropsied piglets, and histological examination confirmed localized intramyocardial hemorrhage without ventricular rupture or pericardial effusion. We propose that incomplete epigenetic reprogramming associated with SCNT, combined with the physiological burden of multi-gene expression, may have compromised myocardial microvascular integrity. Under the abrupt hemodynamic transition that occurs after birth, this structural vulnerability may predispose the left ventricle to intramyocardial hemorrhage and rapid cardiogenic collapse. These findings suggest that left ventricular hemorrhage may represent a previously underrecognized cause of early neonatal death in highly engineered SCNT-derived pigs.

## 1 Introduction

The development of genetically engineered pigs has substantially advanced the field of xenotransplantation by enabling the modification of porcine organs to better withstand human immune responses (1–5). Early strategies focused primarily on preventing hyperacute rejection through the elimination of major porcine xenoantigens, particularly α-1,3-galactosyltransferase, and the introduction of human complement-regulatory molecules (1, 3, 6–8). As xenotransplantation research has progressed, the generation of pigs carrying multiple genetic modifications has become increasingly important for improving graft compatibility and long-term functional survival (2, 4, 5, 8).

Somatic cell nuclear transfer (SCNT) remains one of the most widely used techniques for producing multi-gene porcine models for xenotransplantation. SCNT enables genetically modified donor nuclei to be transferred into enucleated oocytes, allowing the generation of cloned animals carrying complex combinations of knockout and knock-in alleles (6, 7). This approach has been essential for producing pigs lacking porcine xenoantigens while expressing human immune regulatory genes (1, 8).

We previously reported the generation of α-1,3-galactosyltransferase knockout cloned pigs carrying five human immune-related genes: human decay-accelerating factor (hDAF), CD39 (hCD39), tissue factor pathway inhibitor (hTFPI), C1 inhibitor (hC1INH) and TNF-α-induced protein 3 (hTNFAIP3) (9). While the successful genomic integration and expression of these transgenes demonstrated the feasibility of producing complex multi-genic porcine models, a significant challenge emerged: all cloned piglets died within 48 h of birth. Although initial hematological analyses were conducted, they failed to identify a definitive pathological cause of death, leaving the underling mechanisms of this early neonatal mortality unresolved.

Early post-natal mortality is a well-recognized limitation of SCNT-derived animals. Cloned animals frequently exhibit developmental abnormalities, placental dysfunction, abnormal fetal growth, respiratory compromise, and increased perinatal mortality, a constellation of findings commonly referred to as cloning syndrome or large offspring syndrome (10–18). These abnormalities are generally attributed to incomplete epigenetic reprogramming of the donor nucleus during early embryogenesis, which may lead to dysregulated gene expression and abnormal organ development (11, 13, 14, 18, 19). Cardiovascular and vascular abnormalities have also been reported in cloned animals and may contribute to neonatal compromise even in the absence of obvious external malformations (12, 13, 16, 17).

The neonatal period represents a critical stage during which rapid physiological adaptation to extrauterine life occurs, particularly within the cardiovascular system (20, 21). After birth, the circulation rapidly shifts from placental to pulmonary and systemic circulation, exposing the left ventricle to markedly increased systemic vascular resistance (21–23). Structural or functional abnormalities of the myocardium or microvasculature may therefore become clinically significant during this transition, even if they are not apparent at delivery. In addition, neonatal hemostatic regulation differs from that of older animals, which may further influence susceptibility to vascular instability or hemorrhagic lesions during the early post-natal period (24).

According, the present study was designed to conduct a comprehensive pathological investigation into the previously reported cloned piglets. Through detailed gross and histopathological examinations, we identified consistent hemorrhagic lesion localized to the left ventricular myocardium. This follow-up analysis provides critical pathological insight into previously unexplained early neonatal mortality of highly engineered SCNT-derived pigs, highlighting the cardiovascular vulnerabilities that may compromise cloned offspring during the transition to extrauterine life.

## 2 Case description

The genome modified pigs for xenotransplantation were generated in our previous study (Figure 1). In short, to produce the pigs, porcine fetal fibroblasts were genetically modified by targeting for knock-out of porcine genes associated with hyperacute immune responses, α-gal, and knock-in of selected human immune-related genes. Successfully modified cell lines which are no chromosomal abnormalities were detected were used as nuclear donors for SCNT (9). Reconstructed embryos were transferred into synchronized surrogate sows and piglets were delivered and monitored immediately after birth. Clinical status, survival time, and gross abnormalities were recorded.

**Figure 1:**
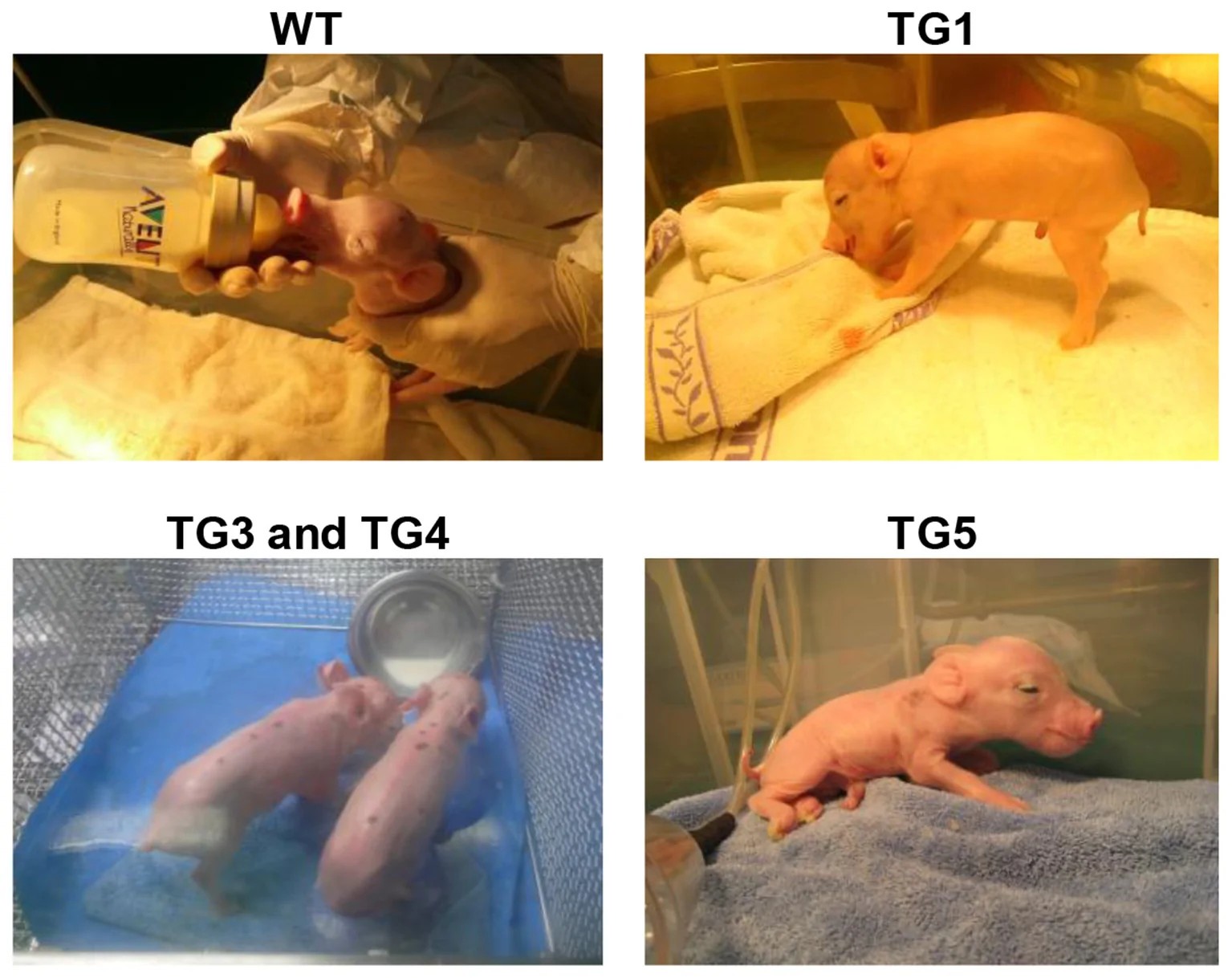
SCNT-derived MGH piglet with knock-out of α-1,3-galactosyltransferase, and knock-in of selected five human immune-related genes (hDAF, hCD39, hTFPI, hC1 inhibitor and hTNFAIP3). WT, non-targeted piglet; TG, targeted piglet.

A total of seven cloned piglets were produced via SCNT. Although no obvious external malformations were observed at delivery, all piglets died within 48 h of birth. Post-mortem examination revealed no evidence of external injury, rib fracture, or thoracic trauma. Major organs, including the lungs, liver, kidneys, arota and gastrointestinal tract, exhibited no consistent gross abnormalities. However, histopathological examination of the cardiac tissues from four piglets revealed consistent hemorrhagic lesions localized to the left ventricular wall. This hemorrhage was primarily confined to the myocardial tissue, with no evidence of associated traumatic injury (Figure 2).

**Figure 2:**
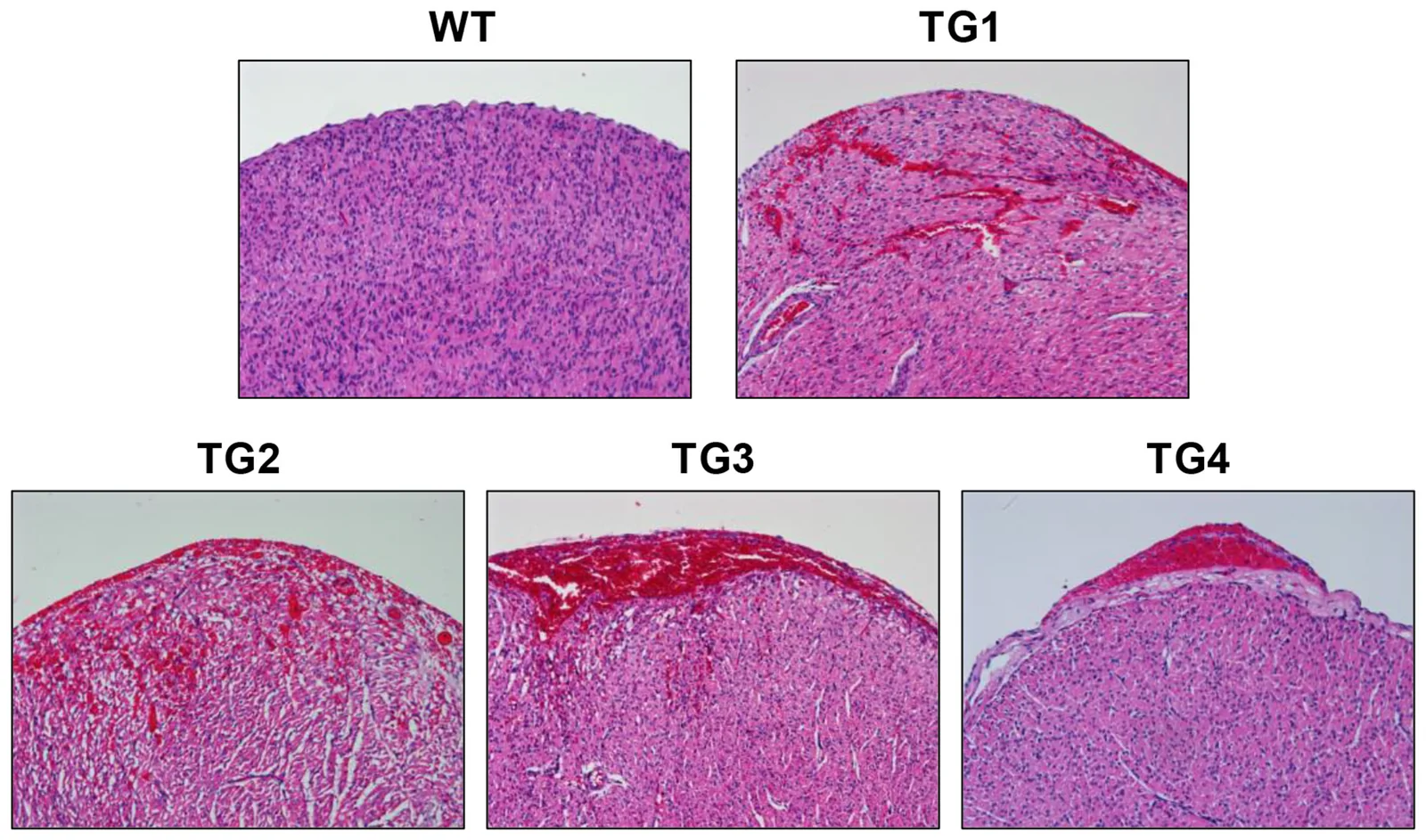
Cardiac tissue collected during necropsy was fixed in 10% neutral buffered formalin, embedded in paraffin, sectioned at 5 μm thickness, and stained with hematoxylin and eosin (H&E). Microscopic examination revealed localized intramyocardial hemorrhage characterized by focal erythrocyte extravasation within the myocardial interstitium. WT, non-targeted piglet; TG, targeted piglet.

## 3 Pathological findings

Complete necropsy was performed on all piglets immediately after death to investigate the cause of early neonatal mortality. The thoracic and abdominal cavities were systematically examined, and major organs including the heart, lung, liver, kidney, spleen, and gastrointestinal tract were examined for gross abnormalities. Particular attention was given to the cardiovascular system.

For histopathological evaluation, representative cardiac tissues were collected during necropsy. Histological sections were examined and interpreted by an experienced veterinary pathologist. Gross necropsy revealed no evidence of external trauma or systemic hemorrhage. The thoracic and abdominal organs did not exhibit consistent abnormalities. However, examination of the heart consistently revealed hemorrhagic lesions localized to the left ventricular wall. The hemorrhage was confined to myocardial tissue and was not associated with ventricular rupture or pericardial effusion.

Histopathological examination confirmed localized intramyocardial hemorrhage characterized by focal erythrocyte extravasation within myocardial interstitial spaces. No inflammatory infiltration, myocardial necrosis, or structural myocardial disruption was observed. In addition, no systemic hemorrhagic lesions were detected in other organs. These finding indicate that the observed lesion represents localized myocardial hemorrhage rather than systemic hemorrhagic pathology.

## 4 Discussion

The present study provides a follow-up pathological investigation of the cloned pigs previously described in our earlier report (9). While our previous work confirmed the successful production of multi-gene pigs, the definitive cause of their death within 48 h remains unsolved. The current findings address this gap by identifying a consistent pathology marker: localized hemorrhagic lesions in the left ventricular myocardium. Notably, necropsy revealed no evidence of systemic trauma or abnormalities in other organs, and histological examination confirmed that the hemorrhage was strictly confirmed to the myocardial tissue without evidence of ventricular rupture or pericardial effusion.

Several interrelated factors may explain this localized pathology. First, developmental disturbances inherent to SCNT must be considered. Animals produced through SCNT frequently exhibit cloning syndrome, which characterized by placental dysfunction and increased perinatal mortality (10, 11, 18), often rooted in incomplete epigenetic reprogramming (14, 15, 19). Although the piglets in this study did not display overt external features of large offspring syndrome subtle defects in myocardial or vascular development, which can be caused in cloned animals (12, 13, 16), may have created a structural vulnerability within the cardiac tissues.

This structural fragility may have been exacerbated by the physiological stress of the transition from fetal to neonatal circulation. During the neonatal period, the heart undergoes rapid cardiovascular adaptation (20, 21). Upon birth, the left ventricle must suddenly overcome undergoes systemic vascular resistance as the placental bypass closes (22, 23). If the myocardial microvasculature is structurally immature or fragile, this abrupt hemodynamic shift could trigger vascular rupture and localized hemorrhage. The specific localization of the lesions to the left ventricle, the chamber bearing the brunt of systemic perfusion, strongly supports this hemodynamic hypothesis.

The previously reported hematological abnormalities in these multi-gene engineered piglets (9), specifically the abnormally low platelet counts (thrombocytopenia), may have established a systemic predisposition to hemorrhage. While systemic thrombocytopenia alone does not account for the distinct localization of the lesions to the left ventricular myocardium, it likely compromised the overall hemostatic capacity of the neonates. This systemic vulnerability, when combined with the abrupt increase in systemic vascular resistance and left ventricular afterload after birth, could have facilitated the focal extravasation of erythrocytes into the myocardial interstitium. Therefore, the observed left ventricular hemorrhage likely represents the convergence of systemic hematopoietic impairment and localized mechanical stress during the critical neonatal transition.

Furthermore, the potential influence of transgene expression warrants scrutiny. The pigs in this study carry five human immune regulatory genes (hDAF, hCD39, hTFPI, hC1INH, and hTNFAIP3) which regulate complement activation, coagulation, and inflammatory pathways (3, 5). For instance, hCD39 expression is known to influence thromboregulation and myocardial responses to injury (4). While these modifications are designed to protect future xenografts, their presence during embryonic development might inadvertently alter vascular homeostasis. However, as specific expression patterns in cardiac tissue were not evaluated in this study, a direct causal link remains speculative.

Taken together, our findings suggest that early neonatal mortality in these genetically engineered pigs is associated with a previously unrecognized cardiac pathology. This localized left ventricular hemorrhage likely reflects a combination of SCNT-related developmental vulnerability, hemodynamic stress during neonatal cardiovascular transition, and potential multi-gene interference. However, several limitations must be acknowledged. The sample size was limited, and detailed cardiovascular functional assessments or molecular analyses of transgene expression in cardiac tissues were not performed prior to death. Further studies involving larger cohorts are therefore necessary to fully clarify these underlying mechanisms.

In conclusion, this follow-up pathological analysis reveals that unexplained neonatal mortality in SCNT-derived pigs may be linked to localized myocardial hemorrhage. These findings highlight the critical importance of comprehensive pathological evaluation and rigorous screening to ensure the viability of complex genetically modified models for future xenotransplantation research.

## Data Availability

The original contributions presented in the study are included in the article, further inquiries can be directed to the corresponding authors.
